# Model agnostic generation of counterfactual explanations for molecules†

**DOI:** 10.1039/d1sc05259d

**Published:** 2022-02-16

**Authors:** Geemi P. Wellawatte, Aditi Seshadri, Andrew D. White

**Affiliations:** Department of Chemistry, University of Rochester Rochester NY USA; Department of Chemical Engineering, University of Rochester Rochester NY USA andrew.white@rochester.edu

## Abstract

An outstanding challenge in deep learning in chemistry is its lack of interpretability. The inability of explaining why a neural network makes a prediction is a major barrier to deployment of AI models. This not only dissuades chemists from using deep learning predictions, but also has led to neural networks learning spurious correlations that are difficult to notice. Counterfactuals are a category of explanations that provide a rationale behind a model prediction with satisfying properties like providing chemical structure insights. Yet, counterfactuals have been previously limited to specific model architectures or required reinforcement learning as a separate process. In this work, we show a universal model-agnostic approach that can explain any black-box model prediction. We demonstrate this method on random forest models, sequence models, and graph neural networks in both classification and regression.

## Abstract 2

Generating model agnostic molecular counterfactual explanations to explain model predictions.

## Graphical abstract

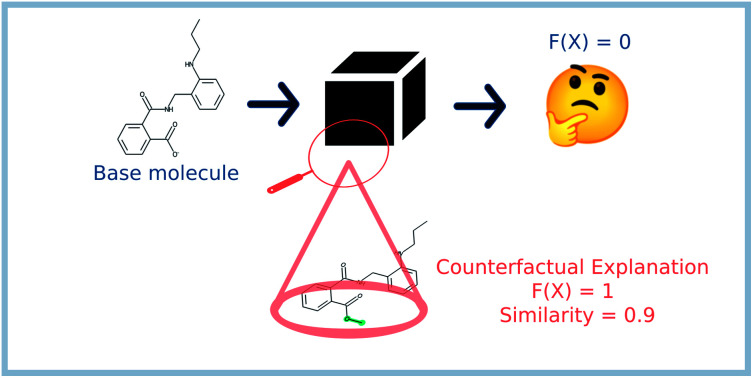

## 1. Introduction

Deep learning has made significant impacts in chemistry because of its ability to regress non-linear relationships between structure and function.^1^ Applications vary from computing quantum properties^2,3^ to predicting chemical properties^4,5^ to screening drug molecules.^6,7^ More specifically, deep neural networks that take in raw graph representations of molecules have proven to be successful when compared with counterparts based on fixed descriptors in both regression and classification tasks.^8^ Despite their empirical accuracy, neural networks are black-box models; they lack interpretability and predictions come without explanation.

Explainable artificial intelligence (XAI) is an emerging field which aims to provide explanations, interpretation, and justification for model predictions. XAI should be a normal part of the AI model lifecycle. It can identify data bias and model fairness.^9^ Users are more likely to trust and use a prediction if it has an explanation.^10^ Finally, it is becoming a legal requirement in some jurisdictions for AI to provide an explanation when used commercially.^11,12^ From a researcher's perspective, XAI can also find the so-called “Clever Hans” effects whereby a model has learned spurious correlations such as the existence of a watermark in images or an over representation of counterions in positive molecule examples.^13^ Despite these benefits of XAI, this is rarely a part of deep learning in chemistry.

Miller^14^ proposes a nomenclature within XAI that distinguishes between a prediction explanation, interpretability of a model, and prediction justification. An explanation is a post-hoc description of why a prediction was made by a model.^15^ Model interpretability is “the degree to which an observer can understand the cause of a decision”.^16^ Finally, justification of a prediction is a description of why a prediction should be believed. Justification typically relies on estimated model generalization error. Interpretable models are common in computational chemistry – DFT, molecular dynamics, and linear regression are inherently interpretable models. Justification is also routine, with almost all recent papers reporting estimated generalization error on withheld test data or from cross-validation. Explanation is rare, especially in deep learning where no insight can be gained by inspecting model weights or parameters.

There are four major approaches for explaining a prediction from a black-box model:^17^ identifying which features contribute the most,^18–22^ identifying which training data contributes the most,^23^ fitting a locally interpretable model around the prediction,^24^ and providing contrastive or counterfactual points.^25^ Feature importance analysis provides per-feature weights that identify how each feature contributed to the final prediction. These can be formulated as SHAP values,^26^ which are a method of computed feature importance weights as a complete explanation (*i.e.*,∑*w*_*i*_ = *f̂*(*x*)).^27^ This is effective when working with a sparse set of molecular descriptors, but when working with thousands of descriptors, SMILES or molecular graphs, this can impart little insight to the human understanding.^14^ A recent study by Humer *et al.*^28^ introduced a model-agnostic visualization tool named CIME for XAI based on feature attribution. Their interactive web-app take in datasets and model predictions to facilitate model interpretation. Authors use SHAP values and Class Attribution Maps (CAM)^29^ to compute feature/atomic attributions in their work. Local interpretable model-agnostic explanations (LIME) provide an implicit “sparsification” relative to feature importance because the locally interpretable model is a different model than the black-box model being explained.^24^ For example, a two dimensional linear regression could be the locally interpretable model. The sparsification arises because we can choose the features going into the locally interpretable model and it can be induced by using regularization when fitting the locally interpretable model to the black-box (*e.g.*, using lasso regression).^30^ Although SHAP values and LIME provide comprehensible explanations, a limitation is that they are not actionable. For example a chemist does not need to know contribution of each feature in a molecule to answer the question “what changes will result in an alternate outcome?”.^31^ This is the motivation behind our approach. We believe this method will be a beneficial tool in real life applications. Therefore, some care must be taken in choosing the locally interpretable model since it needs to fit well around the prediction and must be specifically constructed for the problem of interest.

Counterfactuals are a mature topic in philosophy and mathematics.^32–34^ Reutlinger *et al.*^33^ argue that counterfactual theories can be used to capture scientific explanations of casual and noncasual nature – being more general than causality. Woodward and Hitchcock^32^ define a counterfactual explanation as one that illustrates what differences to an event or instance would generate a change in an outcome. Earliest theoretical definition of counterfactuals was introduced by Kahneman and Miller^35^ in 1986 to explain memory activation to with respect to “what if scenarios”. Counterfactual thinking is now being applied commonly in many fields such as psychology, finance and deep learning.^36–41^ In our work, we use counterfactual explanations to answer “what is the smallest change to the features that would alter the prediction”.^42^ In other words, a counterfactual is an example as close to the original, but with a different outcome. “Your papers would be better cited, if you had a better title”. The example here being a paper identical except the new title and the outcome has changed: the paper is better cited. Furthermore, it can be identified that counterfactual explanations have deep roots in manipulability theories of causation which try to exploit casual relationships for manipulation.^43^ If a process is identified as a manipulation of an event, then there must be a casual relationship between the manipulation and the event.^44^ For example, if the surface contact angle of a droplet of molecules changes when a certain functional group is removed, then we can say that functional group causes the molecule's hydrophilicity.

Another category of explanations is contrastive explanations which explain a prediction by providing related examples of features. Contrastive and counterfactual explanations are once again conceptually similar, but should be distinguished.^25^ In contrastive explanations, one tries to answer “why output X, but not output Y?”^45,46^ rather than “why did output X happen?”. This is similar to recovering the reasoning behind the correct answer of a multiple choice question through the elimination of incorrect options. Contrastive explanations generate explanations by entertaining alternate outcomes whereas a counterfactual explanation shows how to minimally modify our input to get a different prediction.

In the domain of XAI, counterfactuals are intuitive to understand and are sparse because they are as similar to the original prediction as possible.^14,42^ Yet counterfactuals are hard to generate because they arise from optimization over input features – which requires special care for molecular graphs.^47,48^ Namely, molecular graphs are discrete and have valency constraints, making gradients intractable for computation. Here we propose a method that can generate molecular counterfactuals for arbitrary models. These molecular counterfactual provide explanations that are sparse and composed of molecular structures.

**Fig. 1:**
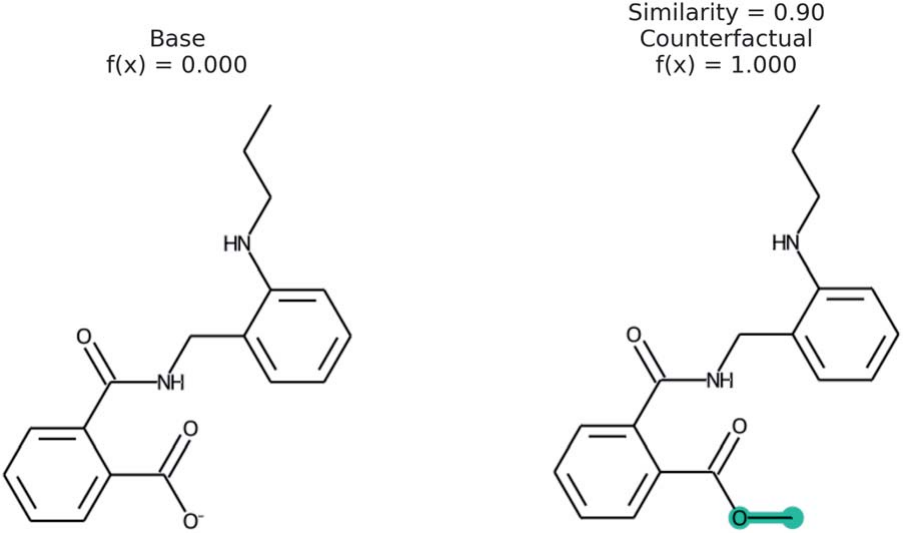
An example of a counterfactual. The molecule on left was predicted to have class of 0, no activity. With the modification shown in teal, the molecule would be in class 1, active. This shows that the carboxylic acid is an explanation for lack of activity.

An example of a molecular counterfactual is shown in Fig. 1. The left molecule is inactive and the right is active. It shows that the carboxylic acid could be made an ester to change activity, giving insight into the reason why the left molecule is not active. The explanation is sparse and intuitive to those with a knowledge of chemical structures. A related concept analogous to counterfactuals is the idea of paired molecules,^49^ where similar molecules with opposite activity are used to understand a class of active compounds. According to Woodward^50^ counterfactuals are only explanations in a space of alternate possibilities. These possibilities help to realize dependencies between initial conditions and outcomes. “They (counterfactuals) do this by enabling us to see how, if these initial conditions had been different or had chanced in various ways, various of these alternative possibilities would have been realized instead”. Therefore, while a counterfactual by itself is sufficient to explain the model, expert knowledge and chemical intuition can strengthen the conclusions.

**Fig. 2:**
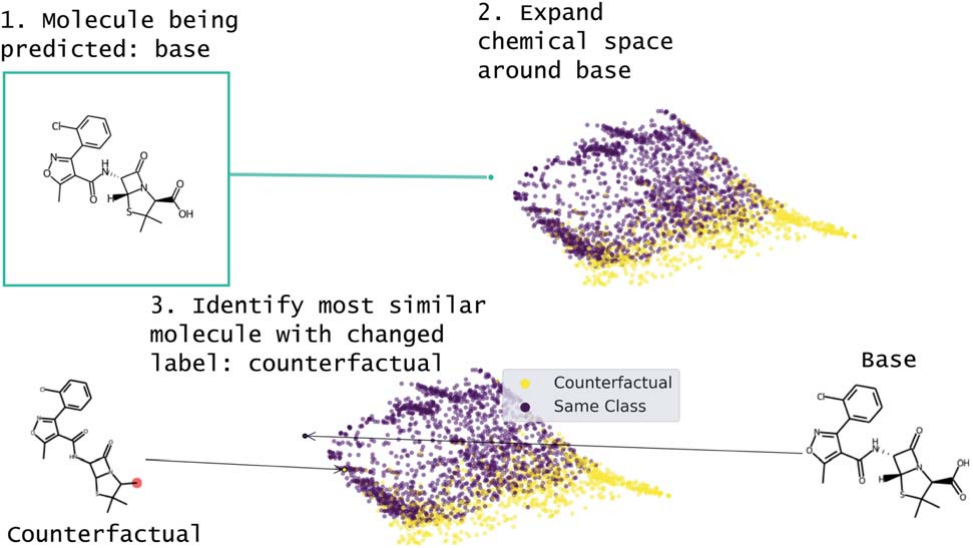
Overview of MMACE. The input is a molecule to be predicted. Chemical space is expanded and clustered. Counterfactuals are selected from clusters to find succinct explanation of base molecule prediction.

Our approach to generating molecular counterfactuals is built on the Superfast Traversal, Optimization, Novelty, Exploration and Discovery (STONED) method which enables rapid exploration of chemical space without a pre-trained generative model or set of reaction rules.^51^ We expand chemical space around the molecule being predicted (base), identify similar molecules with a changed prediction (counterfactuals), and select a small number of these molecular counterfactuals with clustering/Tanimoto similarity. This method works because we represent molecules as SELF-referencIng Embedded Strings (SELFIES) and any modification to a SELFIES is also a valid molecule.^52^ An overview of this process is shown in Fig. 2. Despite SELFIES generating only valid molecules in the sense of satisfied valencies, some of the molecules can involve carbocationic or have unusual rings. Thus we also explore restricting the alphabet of tokens used in STONED. Finally, we propose an alternative approach that obviates this problem by only proposing experimentally available molecules. This method is an enumeration of chemical space around the base molecule by performing a similarity structure search in the PubChem database.^53^

### 1.1 Comparison to existing work

Recent progress in applying XAI methods to graphs, like molecular graphs, is reviewed in Yuan *et al.*^54^ Our method, called Molecular Model Agnostic Counterfactual Explanations (MMACE), produces counterfactual explanations. Counterfactuals are challenging due to the numerical problems associated with both neural networks gradients and working with graph neural networks (GNNs).^55^ There have been a few counterfactual generation methods for GNNs. The counterfactuals-GNNExplanier from Lucic *et al.* uses graph edge operations and a relaxed model prediction function to propose counterfactuals and was found to do well on graph datasets.^47^ Graph edge operations cannot be used on molecular structures because the majority of graph operations will violate valencies. This method also requires model gradients with respect to input, which may not be possible for models outside of neural networks. Our method works on descriptors, graphs, SMILES, and SELFIES features. MMACE does not require gradients, enabling its use on machine learning methods like random forest classification or support vector machines.

Numeroso *et al.*^48^ proposed a molecular explanation generator that is closer to our work. They use a reinforcement learning agent to generate counterfactuals, which ensures that proposed counterfactuals are reasonable molecules. Our method does not require training a counterfactual generator because all molecules resulting from STONED are valid compounds.^51^ This negates the need for a generative counterfactual maker and greatly simplifies the method.

## 2. Theory

A deep learning model takes in as input a set of feature vectors (*x*), and outputs a prediction, denoted as *f̂*(*x*) or *ŷ*. The true value of the property being predicted by the model is denoted as *f*(*x*), or *y*. For chemical applications, *x* is typically a representation of a molecule, which can be a string (SMILES or SELFIES), a set of chemical descriptors, or a molecular graph. Programs including Mordred^56^ and DRAGON^57^ can be used to compute chemical descriptors, such as electronegativity or molecular weight, for each molecule. A molecular graph can consist of a node feature vector and an adjacency matrix. The node feature vector provides information on the type of atoms (*e.g.*, C, H, O, N) present in the molecule and the adjacency matrix provides information on the edges between each node, or which atoms are bonded together.^1^ Together, the node feature vector and adjacency matrix can be used as a molecular graph input to a graph neural network model.^58^

A counterfactual *x*′ is specific to the example of interest *x*, where we have made a prediction *f̂*(*x*). A counterfactual is the explanation of *x* and defined by the solution to the following constrained optimization problem^42^1
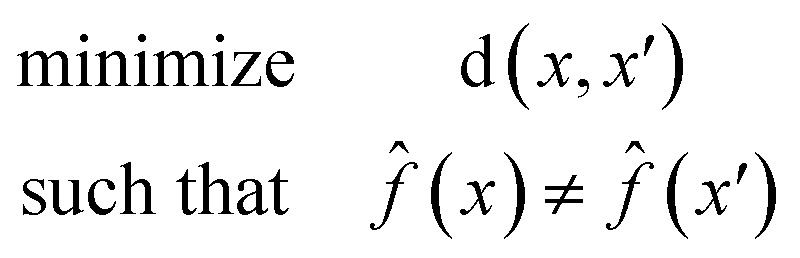
where *x* is the feature vector of our prediction, d(*x*, *x*′) is a measure of distance between features, and *f̂*(*x*) is our model. The counterfactual optimization problem is a function of *x*, so that each time a new prediction is made the counterfactual is also updated.

Eqn (1) is defined for classification tasks. However, this equation must be modified for regression tasks. Instead of finding a conversion in a label, with eqn (2) we find counterfactuals that result in an increase or decrease in the prediction. Here *Δ* is a problem specific hyperparameter which denotes the change in value.2
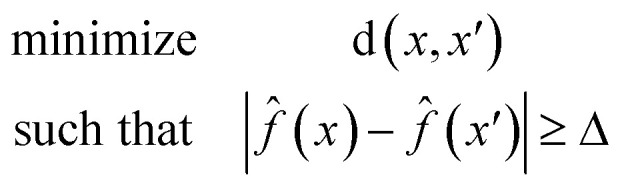


In this work, distance is computed with Tanimoto similarity of ECFP4 molecular fingerprints.^59^ We use Tanimoto similarity as the similarity metric because it is considered the “gold standard” in molecular distance measurements.^60^ Furthermore, Nigam *et al.*^51^ state that impact of fingerprint type in STONED algorithm is minimal as most molecular representations tend to store the same information content.

In principle, this optimization problem could be solved by computing a gradient ∇_*x*_*f̂*(*x*). However, there are complexities of computing gradients with respect to *x* because it may be a molecular graph, a SMILES string, or descriptors which then propagate derivatives to the molecular structure (although see recent progress specifically with SELFIES^61,62^). Instead, previous for counterfactual generation have relied on perturbing *x* using graph transformation operators^47^ and reinforcement learning.^48^ Both these methods have the disadvantage that they can generate chemically infeasible structures, although Numeroso *et al.*^48^ can generate good candidate molecules with sufficient training. Our innovation here is to use the STONED SELFIES method^51^ which rapidly explores local chemical space around a point by exploiting the surjective property of SELFIES: every SELFIES string is a valid molecule. Krenn *et al.*^52^ introduced SELFIES to overcome one of the major limitations in SMILES^63^ that, they do not always correspond to valid molecules. The STONED protocol consists of string insertion, deletion, and modification steps that can generate thousands of perturbations of *x* that are valid molecules and close in chemical space. This requires no training, is independent of features (*e.g.*, molecular graphs, SMILES, descriptors), and requires no gradients.

## 3. Methods

An overview of our method is shown in the schematic in Fig. 2. We use the STONED method as described in Nigam *et al.*^51^ to sample chemical space. Briefly, a starting molecule is encoded into SELFIES and successive rounds of token deletion, replacement, and insertion is done to generate modifications of the starting molecule. This process relies on the surjective property of SELFIES. As in Nigam *et al.*, we limit the number of modifications to the starting SELFIES to ensure we stay local in chemical space. Additionally, starting diversity is improved by exploiting the fact there are multiple non-canonical starting SELFIES. Unless otherwise stated, 3000 modified SELFIES are generated with at most 2 token modifications (mutations). The available tokens (alphabet) for insertion/modification in the STONED algorithm are modified here to use a restricted subset of “intuitive” tokens. Specifically, all positively and negatively charged atoms except O^−^ were removed and the available elements were restricted to B, C, N, O, S, F, Cl, Br, I. We call this the “basic” alphabet. This alphabet can be modified and is discussed further in the results.

RDKit was used for molecule processing, including constructing molecular graphs, drawing molecules, validating input structures, and computing fingerprints.^64^ The scores used in STONED were the Tanimoto similarity^59^ of EFPC4 (ref. 65) fingerprints.

STONED generates a set of molecules around the molecule from which we are predicting (base molecule). To generate counterfactuals, we apply the optimum condition in eqn (1). To generate multiple counterfactuals, clustering is done using DBSCAN^66^ with parameters *ε* = 0.15 and minimum 5 samples per cluster. The distances used for clustering *d* = 1 − *s*, where *s* is pairwise Tanimoto similarity. The most similar molecule from each cluster which satisfies the counterfactual condition is selected and a further reduction by similarity is done if fewer counterfactuals are requested than clusters. DBSCAN infers cluster numbers using the *ε* = 0.15 parameter, which is in units of similarity.

The STONED algorithm does not guarantee the experimental stability of the generated molecules although they are valid (with respect to valency). As an alternative, we use a PubChem similarity search^53^ to populate the chemical space. This approach is similar to STONED method except we query PubChem database rather than generate novel molecules. The same similarity measures are used. This allow us to explore chemical space with only synthetically feasible molecules.

## 4. Experiments

### 4.1 Blood–brain barrier permeation prediction

Predicting if a molecule can permeate the blood–brain barrier is a classic problem in computational chemistry.^67^ The most used dataset comes from Martins *et al.*^68^ It is a binary classification problem with molecular structure as the features. State-of-the-art performance is 0.955–0.988 receiver-operator characteristic area under curve (ROC-AUC) depending on model type and molecular structure featurization.^67^ To test MMACE on this dataset, we developed a random forest model as implemented in Scikit-learn^69^ using molecular descriptors as features. The descriptors are computed with Mordred.^56^ A 20% train/test split was done and the ROC-AUC was computed as 0.91 (see Fig. S1† for ROC curve).

**Fig. 3:**
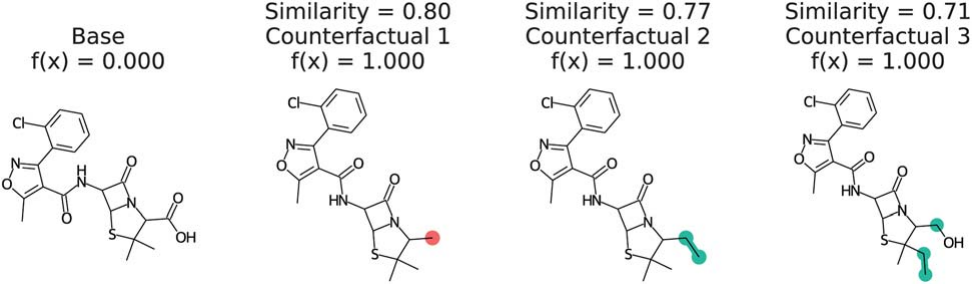
Counterfactual for negative example of blood–brain barrier random forest model. Similarity is computed from Tanimoto similarity of ECFP4 fingerprints.^65^ Red indicates deletion relative to base molecule and teal indicates modification. Counterfactuals show that the removing or modifying carboxylic acid group is the simplest way to make this molecule pass the blood–brain barrier.

Fig. 3 shows a negative prediction from the trained blood–brain barrier classifier. The molecule should not pass the blood–brain barrier. The counterfactuals show what could make the negative example cross the blood–brain barrier, including removing the carboxylic acid (counterfactual 1,3) or changing to an alcohol with additional alkane chains (counterfactual 2). Based on these counterfactuals, the explanation of why this molecule cannot cross the blood–brain barrier is due to the carboxylic acid group. In words: “This molecule will not cross the blood–brain barrier. It would cross the blood–brain barrier if the carboxylic acid were removed”.

### 4.2 Small molecule solubility prediction

Solubility in water plays a critical role in drug design.^70^ Thus, there are many previously developed machine learning tools^47,71,72^ to predict solubility. Solubility is also an intuitive concept that is taught in introductory organic chemistry, thus providing a good setting to test MMACE. We used solubility data from Sorkun *et al.*,^73^ which consists of organic and organometallic molecules. Solubility of the molecule in water is measured in log molarity.

We predict solubility of a given molecule using a gated recurrent unit (GRU) recurrent neural network (RNN)^74^ implemented in Keras.^75^ RNNs are a standard approach in natural language programming tasks because of their ability to handle long sequences and model long-range correlations. Thus, they are commonly used in chemistry applications with SMILES sequences.^76,77^ In our regression model, we use SELFIES because it matches the representation used in MMACE. However, using SELFIES over SMILES does not necessarily translate to better supervised learning performance.^78^

A 10% to 10–80% test–validation–train data split was done. The data, which are specified in SMILES, were canonicalized and converted into SELFIES and training was done for 100 epochs with the Adam optimizer^79^ with a learning rate of 10^−4^. The correlation coefficient on test data is 0.84 and state-of-the-art performance is 0.80–0.93.^80^ Additional model details are listed in the ESI.†

**Fig. 4:**
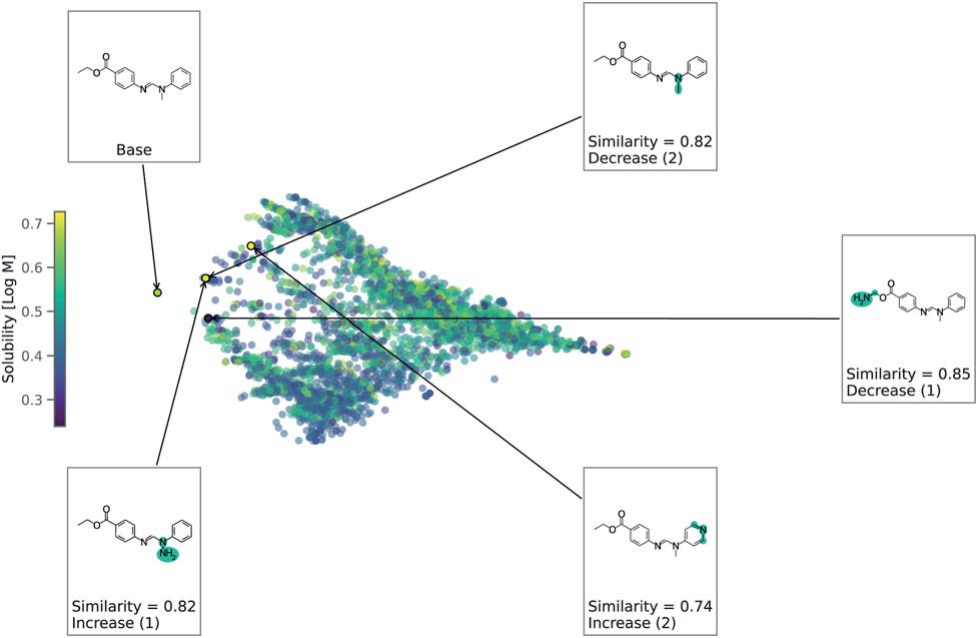
Chemical space for solubility predicting RNN model. This is a principle component analysis of chemical space from Tanimoto similarity distances. Points are colored by solubility. Counterfactuals are annotated.

As this task is regression, we use eqn (2) to account for either an increase or decrease in solubility. We use a value of 1 for *Δ* in eqn (2). Fig. 4 shows counterfactuals generated for a given base molecule. Increase or decrease in solubility is annotated in the counterfactuals. These counterfactuals can be used to explain what functional groups are most important for solubility of the base molecule. According to Fig. 4, the ester, hydrogen bond acceptors, and alkane chain length are contributing reasons for the solubility. The diversity of counterfactuals comes from the DBSCAN clustering, as seen in the principal component analysis projection of chemical space.

### 4.3 HIV activity prediction

Since the first reported case in 1981, the AIDS epidemic has killed 36 million people. According to aid,^81^ currently 1.2 million people in the US have tested positive for HIV (human immunodeficiency virus) which causes AIDS. Although there is no cure for HIV, antiretroviral therapy (ART) reduces mortality and transmission of HIV.^82^ However, effectiveness of ART is limited due to toxicity and cost of treatment.^83^ This means there is still a need for new drugs. Additionally, the National Institute of Allergy and Infectious Diseases has made a systemic study of compounds that can inhibit HIV resulting in large compound datasets. These two facts make predicting potential new HIV drugs a frequently studied task in computational chemistry.^67^

We use a binary classification approach to test MMACE to screen compounds based on their ability to inhibit HIV. The data was downloaded as processed in a Kaggle competition.^84^ This dataset was prepared by the Drug Therapeutics Program (DTP) for AIDS antiviral screening for more than 40 000 compounds.^85^ We use a graph convolutional network (GCN)^86^ implemented in Keras^75^ for molecular featurization and standard dense layers for classification based on molecular features. The inputs to this GCN are the molecular graphs generated with canonicalized SMILES using RDKit software.^64^ However, in the original dataset only 3.5% of the molecules were labeled HIV active. When class imbalances are present, generating counterfactuals for the minor class is easier because the counterfactuals are members of the major class. However, in the alternate case it may require many changes to get a counterfactual and the model may have worse predictive performance on these minor class counterfactuals. Therefore, to address the imbalance between the labels, we used the class weighting technique. A 10% to 10–80% test–validation–train data split was done. The model gains an ROC-AUC of 0.793 after training for only 30 epochs. See Fig. S3 in ESI† for ROC curve. State-of-the-art performance is 0.945–0.993.^87^ For more information on this GCN architecture please refer to ESI.†

**Fig. 5:**
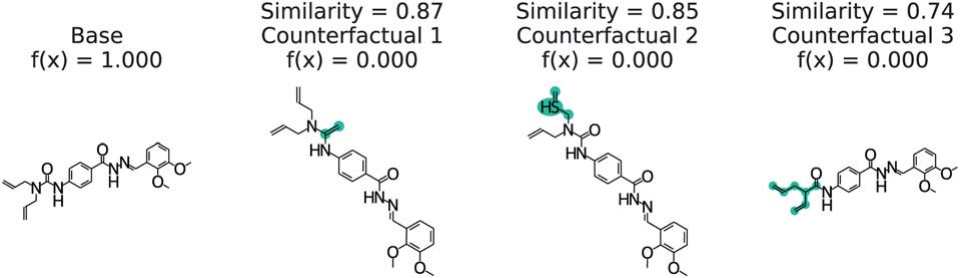
Counterfactuals for positive example of GCN model for classifying HIV activity. Similarity is computed from Tanimoto similarity of ECFP4 fingerprints.^65^ Teal indicates the modifications to the base molecule. Counterfactuals illustrate which modifications make the base molecule HIV active.

Fig. 5 illustrates the top 3 counterfactuals generated from the trained model. The base molecule which is used here is HIV active. Based on the generated counterfactuals, it can be explained that the terminal diamide group has a significant contribution to the HIV activity of this molecule. For example if the terminal amide group is converted to a tertiary amine, then the base molecule will not be active (counterfactual 1). Additional counterfactuals for the same base molecule are provided in the Fig. S4† and reinforce the importance of the diamide group. This shows how chemical reasoning can now be applied to black box predictions through counterfactuals.

### 4.4 Effect of MMACE parameters

There are three main parameters to choose in MMACE: the number of molecules to sample, the number of mutations, and the choice of alphabet. The number of molecules to sample is restricted by the speed of inference of the model being evaluated. Fig. S6† shows that increasing the number of molecules sampled (sample size) increases the number of similar molecules (>rbin 0.7 Tanimoto) as expected, but it begins to saturate after 10 000 samples as duplicates become more common. Based Fig. S6,† we selected a default sample size of 3000 which balances the diversity of chemical space and the number of model inference calls. The models from the experiment section are generally fast enough but majority of time is spent on fingerprint calculation. However, other users of MMACE may have more expensive models and desire fewer samples.

**Fig. 6:**
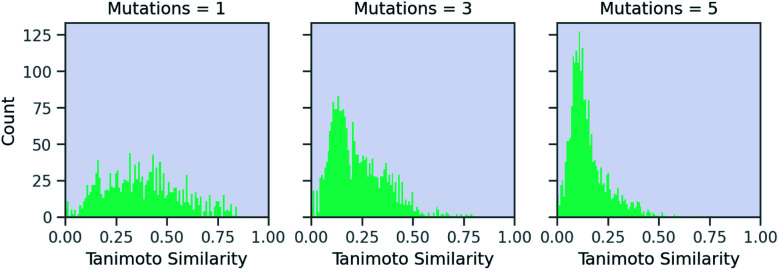
The effect of mutation number on Tanimoto similarity of generated molecules from RNN solubility model. Increasing mutation number reduces number of similar molecules from which counterfactuals can be generated.

Now, we examine the effect of the other two parameters on our RNN model for predicting solubility. There is no direct relationship between number of SELFIES mutations and the similarity. Fig. 6 shows a histogram of molecules arising from STONED as a function of the mutation number from the solubility prediction model. One mutation provides a range of similarities, although few above 0.80 similarity. However, similarity between the base and counterfactuals decreases drastically when the allowed number of mutations increase. Even at three mutations, the majority of molecules are dissimilar and cannot be used for counterfactuals. At five mutations, there are almost no molecules that are comparable with the base molecule. Thus, one and two mutations combined are recommended in MMACE. Fig. S5† illustrates the top counterfactual for a selected base molecule for 1,3,5 allowed mutations. It can be seen that when the allowed mutations are 5, the generated counterfactual molecule is drastically different from the base molecule.

**Fig. 7:**
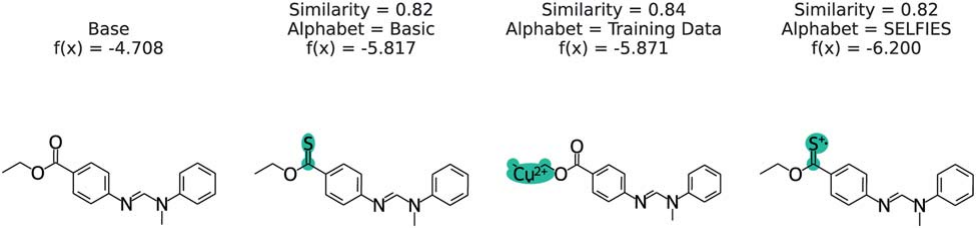
The effect of STONED alphabet choice counterfactuals from RNN solubility model. Although each counterfactual has the same similarity, the molecules are increasingly unusual. The basic alphabet provides a balance of intuitive counterfactuals and enough tokens to explore chemical space.

The effect of the alphabet choice is shown in Fig. 7. Three counterfactuals are shown that are more soluble than the base molecule. In the basic alphabet, recommended for MMACE, we can see that the change to the ester group is reasonable although the carbon–sulphur double bonds are fairly uncommon in nature. In the next example we use the “training data” alphabet which is derived from all unique tokens in the training data. This results in a top counterfactual with a copper(ii) ion. Although the absolute change in predicted label is 1, it provides little understanding about why the original molecule is not more soluble. Finally, the SELFIES alphabet without cation/anions removed can propose counterfactuals simply by ionizing atoms. This does not provide understanding, as these extreme molecules provide little intuition about the base molecule. Although this could be framed as an example of out of distribution predictions, the point of MMACE is to explain predictions and thus we desire an alphabet that results in human interpretable counterfactuals. This is necessarily subjective, but this example shows a limited alphabet provides simpler explanations. Thus, we recommend the basic alphabet in almost all cases. One exception may be organometallic molecules, where exchanging a metal in a counterfactual may be helpful for understanding.

### 4.5 PubChem derived counterfactuals

**Fig. 8:**
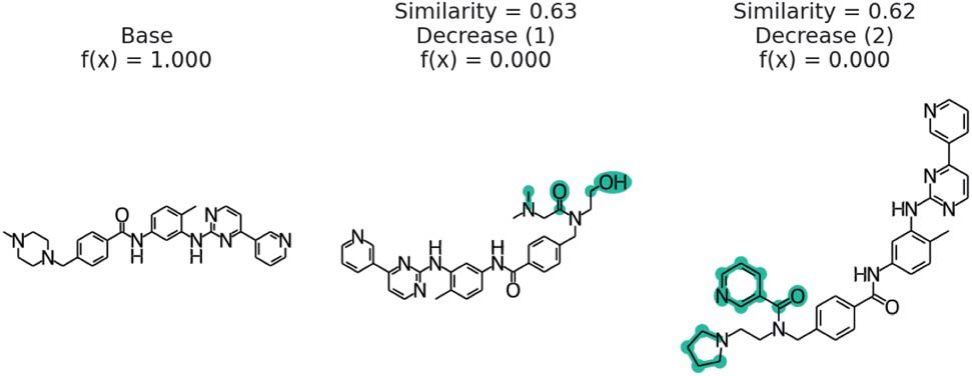
PubChem^53^ derived counterfactuals from the blood–brain barrier permeation prediction.

We examine using PubChem on the blood–brain barrier permeation prediction task with the Gleevec molecule. It is known that Gleevec weakly penetrates the blood–brain barrier.^88^Fig. 8 shows the counterfactuals derived from the PubChem database. The two counterfactuals are structurally similar to the base molecule except the substituted functional groups in the nitrilo group. Based on this result we can conclude the tertiary amine of the pyridine plays a vital role in blood–brain barrier permeation. Although the Tanimoto distance between the base and counterfactuals are higher when compared with STONED method, we are able to generate counterfactuals which are experimentally stable by querying the PubChem database.

## 5. Discussion

Counterfactuals are human interpretable explanations composed of molecular structures that explain model predictions. Counterfactual generation has been a difficult task as it requires feature optimization. The MMACE method overcomes this limitations by enumerating chemical space. Key advantages of MMACE method are that it requires no gradients, training, or additional data to generate per-prediction explanations. Furthermore, MMACE is independent of the model architecture used for classification and regression tasks. Enumerating chemical space was done with the STONED SELFIES method^51^ due to the surjective property of SELFIES.^52^ Furthermore, we explored using the PubChem database to restrictively expand the chemical space with only experimentally feasible molecules during counterfactual generation.

To illustrate the model-agnostic nature of MMACE we test our method on three different model types and three datasets. In the first experiment we use a random forest model which classifies blood–brain barrier permeation of molecules based on the database by Martins *et al.*^68^ In the second experiment we have selected a regression problem that predicts solubility of small molecules using an RNN. Unlike in the previous binary classification experiment which finds counterfactuals with a change in the labels, here we generate counterfactuals which both increase and decrease solubility. In our third experiment, we use a GNN for binary classification of HIV activity of labeled data from the drug therapeutics program.^85^ Furthermore, we have analyzed the effect of three MMACE parameters in counterfactual generation. Based on our findings, we draw the following conclusions; (1) the number of molecules sampled is limited by the inference model while a higher number is better (2) one or two mutations in counterfactuals are recommended (3) the basic alphabet with only B, C, N, O, S, F, Cl, Br, I atoms is recommended.

## 6. Conclusions

AI is causing a seismic shift in chemistry research. Despite the accuracy of AI models, they almost never have interpretations. Thus it can be difficult to understand and trust experiments derived from AI models. This work proposes a universal explainer for any black-box model without requiring training data and regardless of model type. This is based on counterfactuals, which are interpretable explanations composed of molecular structures. To illustrate the model-agnostic nature of MMACE we tested our method on three different model types and three datasets.

## Data availability

All code and data is available at https://github.com/ur-whitelab/exmol.

## Author contributions

ADW and GPW conceptualized the study, curated the data, performed the investigation, did formal analysis, and wrote the manuscript. ADW, GPW, and AS developed methodology and validated the results. ADW acquired funding and supervised the project.

## Conflicts of interest

There are no conflicts to declare.

## Supplementary Material

SC-013-D1SC05259D-s001
