# Supplementary material for: Model agnostic generation of counterfactual explanations for molecules
Source: Chem Sci. 2022 Feb 16;13(13):3697–705. doi: 10.1039/d1sc05259d (PMC8966631; doi:10.1039/d1sc05259d)
Supplement: SC-013-D1SC05259D-s001 [file SC-013-D1SC05259D-s001.pdf]

Electronic Supplementary Material (ESI) for Chemical Science.
This journal is © The Royal Society of Chemistry 2022

Journal Name

ARTICLE TYPE

Cite this: DOI: 00.0000/xxxxxxxxxx

SI: Model Agnostic Generation of Counterfactual Explanations for Molecules

Geemi P Wellawatte[a] Aditi Seshadri,[b] and Andrew D White[b*]

Received Date
Accepted Date

DOI: 00.0000/xxxxxxxxxx

An outstanding challenge in deep learning in chemistry is its lack of interpretability. The inability of explaining *why* a neural network makes a prediction is a major barrier to deployment of AI models. This not only dissuades chemists from using deep learning predictions, but also has led to neural networks learning spurious correlations that are difficult to notice. Counterfactuals are a category of explanations that provide a rationale behind a model prediction with satisfying properties like providing chemical structure insights. Yet, counterfactuals are have been previously limited to specific model architectures or required reinforcement learning as a separate process. In this work, we show a universal model-agnostic approach that can explain any black-box model prediction. We demonstrate this method on random forest models, sequence models, and graph neural networks in both classification and regression.

[a] *Department of Chemistry, University of Rochester, Rochester, NY, USA*
[b] *Chemical Engineering, University of Rochester, Rochester, NY, USA*
[*] E-mail: andrew.white@rochester.edu

RF model

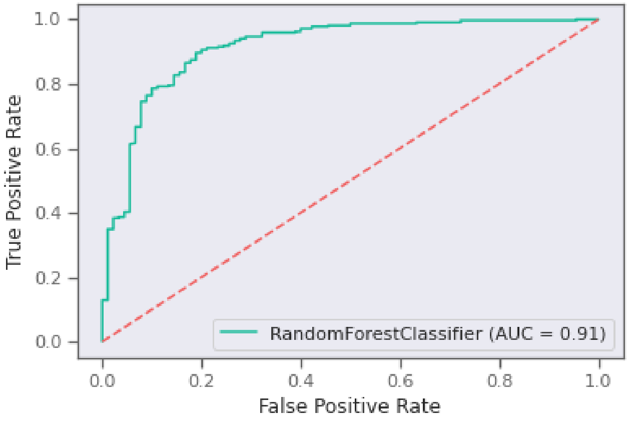

Fig. S1 Random forest model fit to test data with area-under curve analysis of receiver operator characteristic.

RNN model

SELFIES tokens are embedded using a 256 dimensional embedding. The embedded sequence is input to a gated recurrent unit (GRU) RNN.[1] The GRU output goes through one hidden dense layer of dimension 128 with an ReLU activation and a solubility is predicted. The loss is mean squared error in units of solubility, log molarity. The Adam optimizer with a learning rate of 0.01 is used in training.[2] Here, N is a variable which refers to the maximum molecule vocabulary length. Sequences are padded with the "[nop]" SELFIES token. The model fit is shown in Figure S2

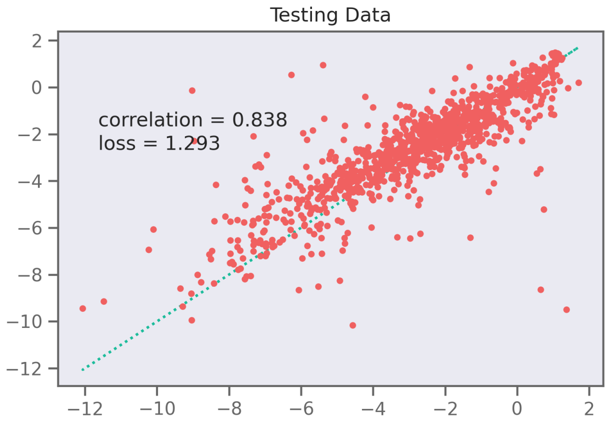

Fig. S2 RNN model fit on testing data. Loss is RMSE.

GCN model

Our GCN model follows the original architecture of Kipf and Welling.[3] Namely, our layer definition is:

Table SI RNN model architecture

Layer type	Shape	Activation
Embedding	(N,256)	None
GRU layer	(N,128)	None
Dense layer 1	(128)	ReLU
Dense layer 2	(1)	None

$$f(V^{(l)}, A) = \sigma\left(\frac{1}{\hat{D}}\hat{A}V^{(l)}W^{(l)}\right) \tag{1}$$

Here, $V^{(l)}$ are the graph level outputs (node features) of layer l. A is the adjacency matrix and $\hat{A} = A + I$ where I is the identity matrix. $\hat{A}$ is used here to add self loops. $\hat{D}$ refers to the node degree matrix. $W^{(l)}$ are the trainable weight matrix for the lth layer.

In our GCN model we stacked 4 graph convolutional layers and 2 dense layers with activation ReLU. The model architecture is shown in table SII. As this is a binary classification task, we use a sigmoid activation in the last dense layer to output predicted HIV activity (HIV inactive: 0 or HIV active: 1). Class weights of 1 and 30 for inactive and active classes are used respectively to address the imbalance in the data. We train our model with binary cross entropy loss and Adam optimizer. A learning rate of 0.01 is used. In this model we have padded the input molecules vectors to be of length 440 (maximum length of molecules in the dataset) to allow batching.

Table SII GCN model architecture

Layer type	Shape (N=400)	Activation
Input 1	(N,100)	None
Input 2	(N,N)	None
GCN layer 1	(N,100)	ReLU
GCN layer 2	(N,100)	ReLU
GCN layer 3	(N,100)	ReLU
GCN layer 4	(N,100)	ReLU
Graph Reduction layer	(100)	None
Dense layer 1	(256)	Tanh
Dense layer 2	(1)	Sigmoid

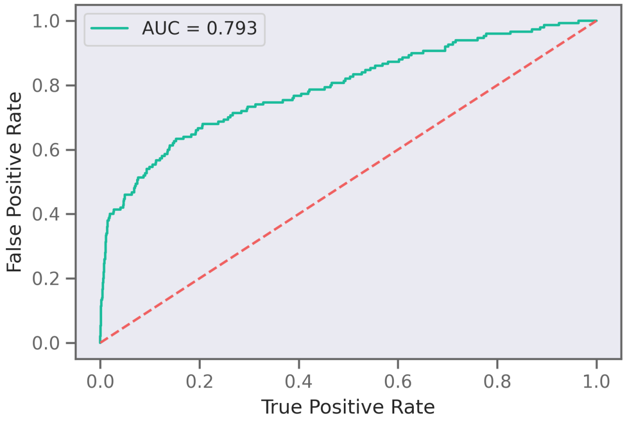

Fig. S3 GCN AUC-ROC plot. Loss is binary cross entropy.

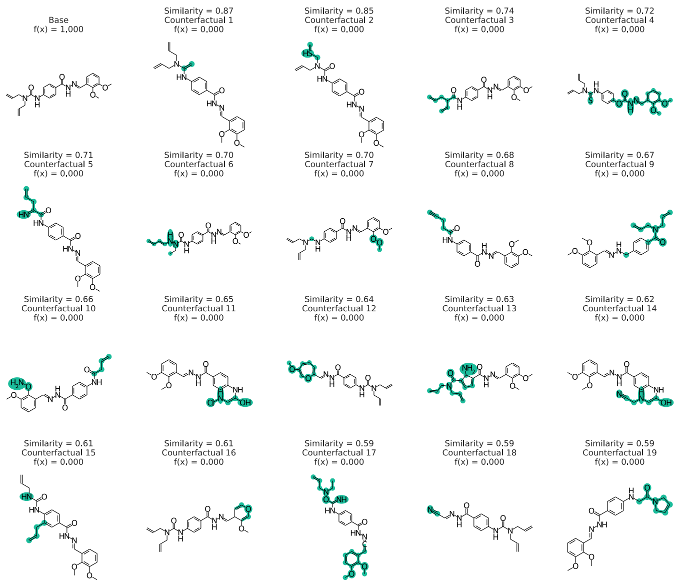

Fig. S4 Additional counterfactuals for the GCN model for predicting HIV activity. Top 19 counterfactuals for the base molecule are illustrated here.

Effect of number of mutations

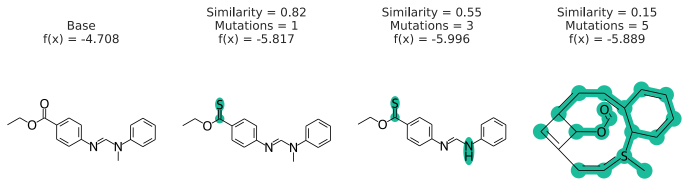

Fig. S5 Top counterfactual for the selected base molecule for each allowed number of mutations. RNN model is used here for the generation of counterfactuals.

Effect of number of samples in the local space

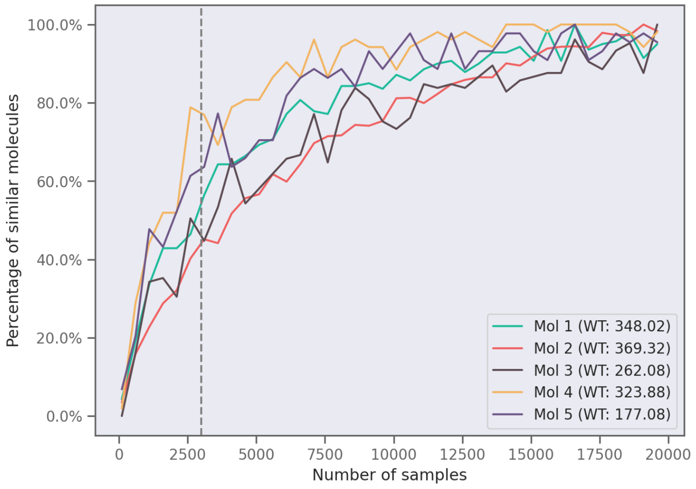

Fig. S6 Percentage of similar molecules as a function of number of samples in the chemical space from RNN solubility model. Percentage is percent of molecules in the generated space with Tanimoto similarity greater than 0.7 relative to the greatest amount observed over whole line (to make curves comparable). The number of similar molecules saturates because there are more duplicates as the sample number increases. The dashed vertical line at 3000 represents the default MMACE parameter. Five randomly selected molecules are illustrated here. SMILES represenations of the molecules are 'CCCCCCOC(=O)C1=CC(I)=C(O)C=C1', 'CCCCCCCC/C=C/CCCCCCCC(=O)N(CCO)CCO', 'c1c(O)C2C(=O)C3cc(O)ccC3OC2cc1(OC)', 'Clc1ccc(Cl)c(c1)c2cc(Cl)c(Cl)c(Cl)c2' and, 'CC(=O)CC(=O)Nc1ccccc1' respectively. Other MMACE parameters: 1 mutation, basic alphabet.

.

Notes and references

1 J. Chung, C. Gulcehre, K. Cho and Y. Bengio, *arXiv preprint arXiv:1412.3555*, 2014.

2 D. Kingma and J. Ba, *International Conference on Learning Representations*, 2014.

3 T. N. Kipf and M. Welling, International Conference on Learning Representations (ICLR), 2017.
